# The Use of Technology for Communicating With Clinicians or Seeking Health Information in a Multilingual Urban Cohort: Cross-Sectional Survey

**DOI:** 10.2196/16951

**Published:** 2020-04-06

**Authors:** Elaine C Khoong, Natalie A Rivadeneira, Robert A Hiatt, Urmimala Sarkar

**Affiliations:** 1 Division of General Internal Medicine Zuckerberg San Francisco General Hospital University of California San Francisco San Francisco, CA United States; 2 Center for Vulnerable Populations Zuckerberg San Francisco General Hospital University of California San Francisco San Francisco, CA United States; 3 Department of Epidemiology and Biostatistics University of California San Francisco San Francisco, CA United States; 4 Hellen Diller Family Comprehensive Cancer Center University of California San Francisco San Francisco, CA United States

**Keywords:** vulnerable populations, health information technology, physician patient relations, consumer health information, digital divide, social media, internet

## Abstract

**Background:**

Technology is being increasingly used to communicate health information, but there is limited knowledge on whether these strategies are effective for vulnerable populations, including non–English speaking or low-income individuals.

**Objective:**

This study assessed how language preferences (eg, English, Spanish, or Chinese), smartphone ownership, and the type of clinic for usual source of care (eg, no usual source of care, nonintegrated safety net, integrated safety net, private or community clinic, academic tertiary medical center, or integrated payer-provider) affect technology use for health-related communication.

**Methods:**

From May to September 2017, we administered a nonrandom, targeted survey to 1027 English-, Spanish-, and Chinese-speaking San Francisco residents and used weighted multivariable logistic regression analyses to assess predictors of five technology use outcomes. The three primary predictors of interest—language preference, smartphone ownership, and type of clinic for usual care—were adjusted for age, gender, race or ethnicity, limited English proficiency, educational attainment, health literacy, and health status. Three outcomes focused on use of email, SMS text message, or phone apps to communicate with clinicians. The two other outcomes were use of Web-based health videos or online health support groups.

**Results:**

Nearly one-third of participants watched Web-based health videos (367/1027, 35.74%) or used emails to communicate with their clinician (318/1027, 30.96%). In adjusted analyses, individuals without smartphones had significantly lower odds of texting their clinician (adjusted odds ratio [aOR] 0.27, 95% CI 0.13-0.56), using online health support groups (aOR 0.14, 95% CI 0.04-0.55), or watching Web-based health videos (aOR 0.31, 95% CI 0.15-0.64). Relative to English-speaking survey respondents, individuals who preferred Chinese had lower odds of texting their clinician (aOR 0.25, 95% CI 0.08-0.79), whereas Spanish-speaking survey respondents had lower odds of using apps to communicate with clinicians (aOR 0.34, 95% CI 0.16-0.75) or joining an online support group (aOR 0.30, 95% CI 0.10-0.92). Respondents who received care from a clinic affiliated with the integrated safety net, academic tertiary medical center, or integrated payer-provider systems had higher odds than individuals without a usual source of care at using emails, SMS text messages, or apps to communicate with clinicians.

**Conclusions:**

In vulnerable populations, smartphone ownership increases the use of many forms of technology for health purposes, but device ownership itself is not sufficient to increase the use of all technologies for communicating with clinicians. Language preference impacts the use of technology for health purposes even after considering English proficiency. Health system factors impact patients’ use of technology-enabled approaches for communicating with clinicians. No single factor was associated with higher odds of using technology for all health purposes; therefore, existing disparities in the use of digital health tools among diverse and vulnerable populations can only be addressed using a multipronged approach.

## Introduction

### Inequities in Technology-Enabled Health Communication Strategies

Technology is increasingly being used to communicate health information [1]. Given the known disparities in internet use and broadband access among rural, older, lower socioeconomic status, nonwhite populations [2], reliance on technology to disseminate health information may exacerbate health inequities [3,4]. In response to the 2009 Health Information Technology for Economic and Clinical Health (HITECH) Act, in the last decade, health systems have joined the broader trend of using technology for communication. As part of the HITECH Act, health systems received financial incentives to provide patients increased access to their health care team and clinical records, measured through patient portal use. Owing to these incentive payments, patient portals have been the primary manner in which health systems and providers have utilized technology for communicating with patients. Unfortunately, studies have consistently shown that patient portals are used less frequently by racial and ethnic minorities, persons of a lower socioeconomic status, and those without neighborhood broadband internet access [5-8].

### Gaps in National Surveys About Health Information Trends

To gain insight on how individuals seek information, the National Cancer Institute (NCI) administers the Health Information National Trends Survey (HINTS) [9]. However, HINTS has several limitations for vulnerable populations; it is distributed only in English and Spanish, lacks the assessment of health literacy, and has poor representation of nonwhite, less affluent populations [10]. As part of an effort to inform local cancer communication strategies, the NCI awarded supplemental funding to Cancer Center Support Grants to administer a modified HINTS tailored for vulnerable populations. In San Francisco, this survey (also known as SFHINTS, San Francisco Health Information National Trends Survey) was administered in English, Spanish, and Chinese to increase data collection from non-English speakers. We also targeted low-income, nonwhite populations who experience cancer outcome disparities (eg, breast and prostate cancer in African Americans and liver cancer in Asians) [11]. Given the proximity to the Silicon Valley, in SFHINTS we included additional questions about the use of technology for exchanging information with a health care professional and the use of social media for health-related purposes.

We previously reported the health information–seeking behaviors and preferences of our SFHINTS cohort and found that participants who preferred English or owned smartphones were more likely to use the internet for health information or prefer emails for provider-distributed health information [12]. To build on these findings, in this report, we explored how both language preference and smartphone ownership impacted the use of technology for communication with health care clinicians or the use of social media for health-related purposes.

### Technology Use for Health Communication in Vulnerable Populations

Despite high levels of interest, prior studies have shown that individuals of a lower socioeconomic status were less likely to use emails to communicate with clinicians [13-17]. However, some studies have shown an increased interest and use in young, nonwhite populations [13,18]. Less is known about other electronic means (eg, SMS text messages and smartphone apps) to communicate with clinicians, particularly among non-English speakers. Similarly, prior studies have shown frequent use of social media for health-related purposes [19-21], but studies have rarely included non–English speaking, low socioeconomic populations.

Given the local nature of SFHINTS, we were able to explore an additional contributor to participants’ use of technology for clinician-directed communication: the type of clinic that participants use for their usual source of care (eg, safety net clinic or academic tertiary medical center). Prior literature has shown usability challenges for electronic health records (EHRs) [22] and that safety net EHRs are less likely to have patient engagement features (including patient portal–related features), potentially exacerbating inequities in patient-clinician communication [23,24]. Consequently, safety net clinics that disproportionally serve groups experiencing health disparities may also lack the technology infrastructure to facilitate technology-based approaches to increase communication and access to health care clinicians.

The multilingual, vulnerable population in the SFHINTS cohort provided an opportunity to explore the impact of three relatively understudied factors—language preference, smartphone ownership, and type of clinic for usual care (eg, safety net clinic or academic tertiary medical center) as a proxy for the digital infrastructure or patient portal usability—on participants’ use of technology to communicate with their health care team and participants’ use of social media for health-related purposes. We hypothesized that language preference and smartphone ownership would impact all technology use, but type of clinic would only impact technology use for communication with clinicians.

## Methods

### Research Setting

The 2017 American Community Survey estimates that San Francisco has a minority-majority: 34% Asians, 15% Latinx, and 5% African American. Nearly 45% of residents speak a non-English language, with Chinese being the most common; one-fifth of San Francisco residents have limited English proficiency [25]. There are several health care systems in San Francisco that deliver primary care, and each system uses its own EHR system. There is one tertiary academic center (University of California San Francisco) as well as two integrated payer-provider systems (Kaiser and Veterans Affairs). The primary care clinics in these systems have used EHR systems with English-language patient portals for over 5 years. There are two larger networks of safety net clinics within San Francisco. One group of clinics, which uses the same EHR, is run by the Department of Public Health, which also operates the county hospital. The patient portal within these clinics had been active for approximately 2.5 years at the time of survey administration and was only available in English. The other group of safety net clinics is a consortium of loosely affiliated clinics. Each clinic has independently chosen an EHR system and, therefore, the patient engagement features of the EHR at each clinic are variable. Similarly, the remaining private and community clinics within San Francisco vary in terms of the EHR system and availability of patient access or engagement features. At the time of the survey, only Kaiser offered a non–English language patient portal (Spanish).

### Survey Development

We used English and Spanish HINTS questions [9] as well as validated health care access [26] and health literacy [27] questions to create our survey (SFHINTS: Multimedia Appendix 1, questions relevant to this report are in sections B-D, G, and H.) We used a standard dual-reviewer process [28] to translate questions for the Spanish and Chinese surveys. The details of survey development and administration are described in prior papers [29].

### Sampling Procedure, Recruitment, and Survey Administration

Using community-based snowball sampling with prespecified language and race and ethnicity targets to reach populations with known cancer disparities, we aimed for half of the surveys to be completed in English (with half of the participants identifying as African American) and non-English surveys to be equally divided between Spanish and Chinese (Mandarin or Cantonese) participants [11,29].

As previously reported in greater detail [12,29], from May to September 2017, bilingual staff administered the survey in-person on tablet devices at community establishments and events as well as small businesses and street locations in specific neighborhoods to target our populations of interest. (Surveys were administered via REDCap [Research Electronic Data Capture] electronic data capture tools hosted at our institution. REDCap [30,31] is a secure, Web-based software platform designed to support data capture for research studies.) The staff explained the survey’s purpose, acquired verbal consent, and then administered the survey in the participants’ preferred language. A US $25 incentive was provided; our institution’s institutional review board approved this study.

### Conceptual Model and Predictor Variables

We used an information-seeking behavior and use model described by Longo (Textbox 1) to identify a complete list of potential predictors that could explain the variation in participants’ use of technology for health purposes [32]. Longo [32] described both contextual and personal factors that impact behavior. The SFHINTS survey included more personal than contextual factors. Ultimately, we included a total of 10 predictor variables. We had three predictor variables of primary interest: two contextual factors—smartphone ownership (an information environment factor) and type of clinic (a health care structure)—and one personal factor (ie, language preference). Guided by prior literature, we included seven additional personal factors (ie, age, gender, race or ethnicity, health literacy, education, English proficiency, and current health status) as predictors of technology use for health purposes [33-36].

Smartphone ownership was a binary variable (ie, yes vs no). We also dichotomized health status (ie, poor or fair vs good or very good or excellent), English proficiency, and health literacy. English proficiency and health literacy were reported as limited if participants reported speaking English less than *well* or if participants felt less than *quite a bit* comfortable completing medical forms independently [27]. Within the race or ethnicity and language variables, non-Hispanic white and English served respectively as the reference categories for analyses. We categorized age (ie, 18-34 as the reference category, 35-49, 50-64, and ≥65 years) and education (ie, less than high school; high school or equivalent; some college or vocational training; at least college graduate as the reference category) into four groups. Clinic type was organized into six categories: no usual source of care (reference category), nonintegrated safety net, integrated safety net, private or community clinic, tertiary academic, and fully integrated payer and provider (Kaiser Permanente or Veterans Affairs).

**A conceptual model of the factors that impact information-seeking behaviors.**

**Contextual**

- Health status
- Health care structure
- Delivery of care
- Information environment factors
- Information seeking for self, family, or friend at risk or with a current medical problem

**Personal**

- Demographic factors
- Socioeconomic factors
- Health history
- Family medical history
- Education
- Culture
- Languages
- Attitudes, intentions, behaviors
- Current health status

### Outcome Variables

We reported the use of technology for health communication with clinicians or peers (questions B4 and B5 in the SFHINTS Survey in Multimedia Appendix 1) as a binary variable (yes or no) for the five sources used by at least 9.5% of participants (ie, use of an email, an SMS text message, or an app with a health care provider and use of online support groups or health-related videos). Each survey respondent was able to answer yes or no for each type of technology use. On the basis of prior literature showing differences in the use of these types of technology, we reported these as independent outcomes rather than an aggregated outcome [13,18]. Therefore, five different regression models have been reported for outcomes in this report.

### Analysis

The relationships between predictor variables and technology use were assessed using bivariate logistic regressions. In addition, we conducted weighted, multivariable logistic regression analyses to identify factors associated with technology use for each of the five outcomes. Weights were computed using iterative proportional fitting (raking). This technique is used for nonprobability samples and involves iteratively adjusting over a set of variables (ie, age, gender, and education) within each race or ethnicity group to reweight the respondent population to match the distribution of the reference population (ie, San Francisco) [37]. We determined no significant collinearity (tolerance >0.10) between predictor variables. All logistic regressions were done using the PROC SURVEYLOGISTIC procedure of SAS 9.4 statistical software (Cary, North Carolina). All regressions were performed using complete case analyses, which totaled 944 observations (944/1027, 91.92% of respondents). The data analyzed for this study is available from the senior investigators of the SFHINTS study (RH and US).

## Results

### Participant Characteristics

The 1027 participants (514 English surveys with 242 non-Hispanic black, 115 Latinx, and 43 non-Hispanic white participants; 256 Spanish surveys; and 257 Chinese surveys) have been previously described (Multimedia Appendix 2: participants’ sociodemographic traits) [12,29]. Our cohort had more limited English-proficient participants (344/1027, 33.50%) than the 2017 national HINTS cohort (2%) [9]. In our cohort, 440/1027 (42.84%) participants had limited health literacy, whereas 791/1027 (77.02%) owned smartphones. Nearly one-fifth (178/1027, 17.33%) reported no usual source of care. Over 50% (148 nonintegrated safety net clinics and 378 integrated safety nets) received care in the safety net systems.

### Use of Technology

**Figure 1:**
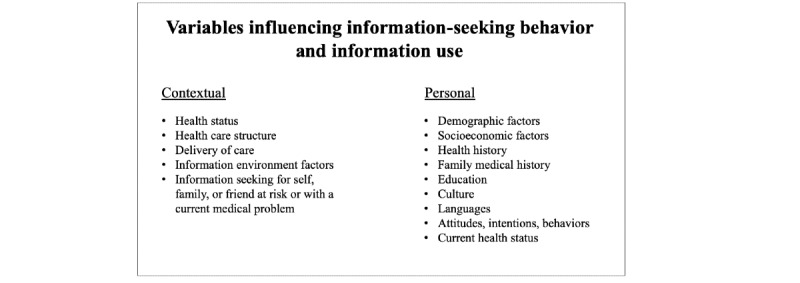
The use of technology for health purposes by smartphone ownership and language preference. Percentage is based on the subset of participants without smart phones (N=236) or with smartphones (N=791) for the left and right panel respectively.

As detailed in Table 1, approximately one-third (318/1027, 30.96%) of the participants used an email to communicate with clinicians. Fewer used an SMS text message (218/1027, 21.23%) or an app (136/1027, 13.24%) for communications with clinicians. The use of Web-based videos to learn about health information was common (367/1027, 35.74%). Across all language groups, at least one-quarter of the population reported watching Web-based videos about health. Online support groups were used by the lowest portion of respondents (99/1027, 9.64%). Figure 1 shows the use of technology by language and smartphone ownership. Smartphone owners across all language preferences had higher rates of using all forms of technology.

Table 2 reports results of the bivariate logistic regression analyses for the three predictors of interest. In the unadjusted analyses, all 10 variables impacted the odds of using at least one form of technology (see Multimedia Appendix 3). For the three predictors of particular interest in our study, in unadjusted analyses, individuals without smartphones had lower odds of using all forms of technology. We also found that individuals who preferred Spanish had lower odds of using an email to communicate with clinicians, whereas individuals who preferred Chinese had lower odds of using an email, an SMS text message, or apps to communicate with clinicians as well as watching Web-based health videos. Relative to respondents with no usual source of care, participants who received care at an integrated safety net, academic tertiary medical center, or integrated payer-provider clinic had higher odds of using technology (ie, emails, SMS text messages, or apps) to communicate with clinicians. Respondents who received care at a private clinic or an integrated payer-provider clinic had higher odds of watching Web-based health videos.

In the multivariable analyses, when holding all other variables constant, race or ethnicity, English proficiency, and health literacy no longer significantly predict any of the outcomes, but younger females with more education had higher odds of using at least one form of technology (adjusted odds for all variables are in Multimedia Appendix 4). Of the three variables of primary interest for this report, we found that individuals without smartphones had lower odds of using text to communicate with clinicians, joining online support groups, or watching Web-based health videos (Table 3). Individuals who preferred Chinese had lower odds of using SMS text messages to communicate with clinicians, whereas Spanish-speakers had lower odds of using apps to communicate with clinicians and joining online support groups. Individuals who received care at an integrated safety net, academic tertiary medical center, or integrated payer-provider clinic had higher odds than respondents with no usual care of using emails, SMS text messages, or apps to communicate with clinicians. Respondents that received care at the 2-clinic systems which adopted patient portals the earliest in San Francisco (academic tertiary system and integrated payer-provider) had higher odds of using apps to communicate with clinicians. Participants with care at the private clinic or community hospital had higher odds of watching Web-based health videos.

**Table 1:** The use of technology to communicate with clinicians or the use of social media for health purposes.

Types of technology use		All (N=1027), n (%)	English (n=514), n (%)	Spanish (n=256), n (%)	Chinese (n=257), n (%)
**Technology use to communicate with clinicians**					
	Email	318 (30.96)	199 (38.7)	76 (29.7)	43 (16.7)
	SMS text message	218 (21.23)	142 (27.6)	56 (21.9)	20 (7.8)
	App	136 (13.24)	92 (17.9)	29 (11.3)	15 (5.8)
**Social media use for health purposes**					
	Online support group	99 (9.64)	59 (11.5)	19 (7.4)	21 (8.2)
	Web-based health videos (eg, YouTube)	367 (35.74)	189 (36.8)	109 (42.6)	69 (26.8)

**Table 2:** Unadjusted odds of using technology for health purposes.

Predictor		Email with clinician, uOR^a,b^ (95% CI)	SMS with clinician, uOR^a^ (95% CI)	App with clinician, uOR^a^ (95% CI)	Support group, uOR^a^ (95% CI)	Web-based videos, uOR^a^ (95% CI)
No smartphone		0.25 (0.12-0.52)^c^	0.41 (0.17-0.99)^c^	0.325 (0.13-0.84)^c^	0.10 (0.04-0.26)^c^	0.14 (0.07-0.25)^c^
**Language^d^**						
	Spanish	0.48 (0.27-0.85)^c^	0.85 (0.46-1.57)	0.51 (0.25-1.04)	0.46 (0.20-1.06)	0.76 (0.42-1.36)
	Chinese	0.29 (0.17-0.49)^c^	0.23 (0.12-0.44)^c^	0.22 (0.10-0.47)^c^	0.64 (0.29-1.42)	0.52 (0.32-0.84)^c^
**Type of clinic for usual source of care^d^**						
	Nonintegrated safety net	1.10 (0.43-2.80)	0.56 (0.25-1.27)	0.74 (0.27-2.00)	0.54 (0.19-1.52)	0.91 (0.42-1.96)
	Integrated safety net	2.03 (1.04-3.98)^c^	3.26 (1.44-7.38)^c^	1.11 (0.44-2.83)	0.83 (0.27-2.50)	1.59 (0.83-3.05)
	Private clinic or community hospital	1.15 (0.45-2.94)	0.69 (0.26-1.83)	1.17 (0.42-3.29)	0.37 (0.11-1.25)	2.85 (1.13-7.19)^c^
	Academic tertiary medical center	14.10 (4.58-43.38)^c^	4.54 (1.03-20.02)^c^	9.09 (2.05-40.28)^c^	1.36 (0.21-8.72)	3.51 (0.90-13.72)
	Integrated payer and provider	4.46 (1.71-11.64)^c^	0.83 (0.30-2.28)	3.92 (1.22-12.58)^c^	2.14 (0.57-8.08)	2.66 (1.10-6.43)^c^

^a^All odds ratios are weighted but unadjusted within this table.

^b^uOR: unadjusted odds ratio.

^c^*P*<.05.

^d^The reference categories for the following variables are as follows: language (English), type of clinic (no usual source of care).

**Table 3:** Adjusted odds of using technology for health purposes.

Predictor		Email with clinician, aOR^a,b^ (95% CI)	SMS with clinician, aOR^a^ (95% CI)	App with clinician, aOR^a^ (95% CI)	Support group, aOR^a^ (95% CI)	Web-based videos, aOR^a^ (95% CI)
No smartphone		0.61 (0.25-1.48)	0.27 (0.13-0.56)^c^	1.12 (0.42-2.99)	0.14 (0.04-0.55)^c^	0.31 (0.15-0.64)^c^
**Language^d^**						
	Spanish	0.69 (0.28-1.69)	0.51 (0.16-1.62)	0.34 (0.16-0.75)^c^	0.30 (0.10-0.92)^c^	0.66 (0.21-2.02)
	Chinese	0.97 (0.41-2.30)	0.25 (0.08-0.79)^c^	0.32 (0.10-1.03)	2.01 (0.50-8.06)	1.00 (0.41-2.41)
**Type of clinic for usual source of care^d^**						
	Nonintegrated safety net	1.02 (0.39-2.66)	0.64 (0.25-1.63)	1.12 (0.36-3.54)	0.55 (0.17-1.81)	0.68 (0.27-1.69)
	Integrated safety net	2.36 (1.08-5.12)^c^	2.96 (1.25-7.04)^c^	1.60 (0.57-4.54)	0.47 (0.14-1.57)	1.35 (0.65-2.83)
	Private clinic or community hospital	1.02 (0.33-3.15)	0.72 (0.22-2.33)	1.41 (0.31-6.42)	0.19 (0.03-1.08)	2.65 (1.08-6.51)^c^
	Academic tertiary medical center	9.08 (2.46-33.5)^c^	3.39 (0.57-20.19)	12.41 (2.76-55.89)^c^	0.70 (0.11-4.28)	2.50 (0.75-8.29)
	Integrated payer and provider	2.68 (0.99-7.27)	0.78 (0.27-2.26)	4.81 (1.44-16.05)^c^	1.20 (0.30, 4.80)	1.79 (0.74-4.32)

^a^All odds ratios are weighted and adjusted for age, gender, race or ethnicity, English proficiency, education, health literacy, health status, smartphone ownership, language preference, and type of clinic for usual source of care.

^b^aOR: adjusted odds ratio.

^c^*P*<.05.

^d^The reference categories for the following variables are as follows: language (English) and type of clinic (no usual source of care).

## Discussion

### Principal Findings

Even in vulnerable, diverse populations, nearly 4 in 5 individuals own a smartphone, with a large portion using mobile technologies to communicate with clinicians. Just over one-third of individuals are watching health-related videos online, including over 1 in 4 participants regardless of language preference. For the most part, many of our findings are consistent with prior literature that females who are younger and better educated are more likely to use digital tools to engage in their health care [13,16,36]. Importantly, no variable was a significant predictor for all five technology use outcomes, which highlights the importance of distinguishing among different types of digital tools when devising communication strategies for diverse populations. Digital communication strategies may need to be tailored to reach one specific population versus a different population.

Although we had anticipated that smartphone ownership and English language preference would be associated with higher odds of using all types of technology, both factors were significantly associated with only a subset of the outcomes. We had also anticipated that type of clinic should only be associated with the use of technology to communicate with clinicians but found that it was also associated with whether respondents watched Web-based health videos. Unfortunately, we do not have data to explore potential explanations for these findings. However, we can use the technology acceptance model (TAM) [38] as a conceptual model to try to explain why certain populations may be more likely to use technology for each purpose. In the TAM, whether or not an individual adopts a technology is impacted by two main factors: perceived ease of use or perceived usefulness of a given technology.

### Smartphone Possession Alone Does Not Increase the Use of All Types of Technology

For example, smartphone ownership was a significant predictor only for the use of online support groups, Web-based health videos, and SMS text messaging with clinicians. Using TAM to guide our thinking, we can hypothesize that smartphone owners perceive a greater ease (or usefulness) of only texting a clinician, watching an online health video, or using an online support group. Smartphone ownership was not a significant predictor of using emails or apps with clinicians perhaps because the perceived ease or usefulness of these activities was not as different between smartphone owners and nonowners. We do not have data to assess this perception and propose that future research in digital health equity should explicitly evaluate how provision or ownership of a smartphone alone impacts the use of digital tools to engage with clinicians or Web health resources.

Another potential explanation is that not all smartphone owners have the digital literacy to use all the functions of the smartphone. Studies have shown that digital health literacy, a distinct concept from health literacy, poses a barrier for using digital health tools in underserved populations [39]. A digital equity survey of more than 1000 San Francisco residents found that although 93% owned smartphones, 5% did not have a data plan. Among those with an income less than US $25,000, 79% owned smartphones, but 14% of these smartphone users did not have a data plan [40]. Without data access, users are inherently limited in the number of activities that can be performed on their smartphones. This same survey also found that nearly 25% to 30% of internet users who were non–English speaking, older than 65 years, or had an income less than US $25,000 did not possess basic digital literacy (defined as the ability to search for information, find a website, send an email, or fill out an online form) [40]. Digital literacy may be a mediator or moderator, which explains why smartphone ownership was not found to be an important predictor for all technology outcomes.

Surprisingly, smartphone ownership was not an important predictor of using apps to communicate with clinicians, despite apps necessitating the ownership of a smartphone or tablet. Our survey did not allow us to explore if respondents answered this question while envisioning a patient portal app or an alternative communication app (ie, Facebook messenger, WhatsApp, etc). Regardless, these findings suggest that the apps currently available for communicating with clinicians are not adequately useful or easy to use (TAM constructs) such that smartphone owners are more likely to use apps than nonsmartphone owners. If participants were considering patient portal apps when answering these questions, it further supports assertions that health care system digital communications are not mobile friendly [41].

### Language Preference Predicts Technology Use Patterns Beyond English Proficiency

We also anticipated that language preference, similar to smartphone ownership, would be associated with all technology use behaviors given the known barriers to communicating with health care clinicians for limited English-proficient patients as well as the higher quantity of English-language health content in the internet [42-44]. Although English proficiency and language preference are often correlated, they are distinct concepts [45,46]. Specifically, studies have found language preference to be associated with acculturation even after accounting for English proficiency measures, and acculturation has been found to impact health information–seeking behaviors [34,47-49].

With this in mind, it is worth noting that English proficiency was not a significant predictor for any of the studied outcomes. On the contrary, both Spanish and Chinese preferences were associated with lower odds of using at least one form of technology. Of note, neither using emails to communicate with clinicians nor watching Web-based videos was significantly impacted by language preference. These findings may suggest that the usefulness or ease of using emails or Web**-**based health videos is not significantly different for individuals who prefer English vs non-English languages and, therefore, that both these approaches may be potential avenues for communication, which will avoid significantly exacerbating communication disparities already experienced by non-English speakers. Although Web-based health videos have been found in some studies to be an effective means of disseminating information to non–English speaking populations [50-52], earlier studies have suggested differences in email use based on the language (though earlier studies did not consider both English proficiency and language preference) [24].

### Health System Factors May Impact Electronic-Based Communication With Clinicians

Owing to differences in the digital and EHR infrastructure within different San Francisco health care systems, we anticipated that the type of clinic would impact behaviors surrounding communication habits with clinicians. Moreover, as the reference variable was no usual source of care, we anticipated that having any usual source of care should result in higher odds of communicating with clinicians. We did find that respondents who received usual care at clinics affiliated with the academic tertiary medical center or the integrated payer-provider health care systems—systems that have had the longest, most established patient portal systems—had higher odds of using apps to communicate with clinicians, potentially through more mature patient portal apps. Notably, care at either of the safety net systems or private clinics not clearly affiliated with large health care systems was not associated with higher odds of using apps. This supports the literature that safety net EHRs are less likely to have usable patient engagement features [23]. It also reinforces findings from a recent study that found patients at safety net systems are less likely to use a patient portal, and patients receiving care at nonacademic medical centers and small health care systems are less likely to access their medical records [53].

The odds of emailing your clinician were higher for individuals whose primary source of care was a clinic affiliated with the integrated safety net or an academic medical center. One possible explanation for this is that many of the clinicians who provide care in the integrated safety net are faculty at the main academic medical center in San Francisco. There may be behaviors or attitudes about patient engagement that are common to these group of clinicians which results in their patients perceiving higher ease or usefulness of emailing their clinician. This is similar to patient portal usage studies that show patient-clinician relationships and clinician attitudes and behaviors about patient portals impact their patients’ use of the patient portal [54,55].

### Limitations

This study is limited by its reliance on participant self-reporting and sampling of a single city and county, which was inherent in the design to inform local communication efforts. However, we surveyed more than 1000 individuals from groups underrepresented in health information–seeking studies. We did not collect information on who declined to participate in the survey and, therefore, could not report a response rate. This may have resulted in a sampling bias, but weighting our sample should reduce bias, and in-person surveys generally show higher response rates than other survey methods [56]. A small number of observations within the levels of independent variables (eg, college graduates and patients at academic tertiary medical care clinics) resulted in some estimates with wide confidence intervals.

### Conclusions

We found that even after controlling for other known factors, smartphone ownership, language preference, and type of delivery care system for usual care changed the odds of using technology for health purposes. Smartphone ownership was important for some behaviors and, therefore, ensuring technology and communications are optimized for mobile devices is important. However, none of the studied patient or contextual factors was significantly associated with all behaviors, suggesting that health care systems and public health messages may have to utilize a variety of approaches when using technology-enabled communications to reach a broad population. These communication strategies must be delivered with an eye on equity for diverse, underserved populations to ensure any intervention does not exacerbate the existing disparities. No single solution alone—including the provision of smartphones, creation of non-English communications and workflows, or development of better patient engagement digital infrastructure—is likely to fully address the existing inequities in the use of digital health tools.

## Appendix

Multimedia Appendix 1 San Francisco Health Information National Trends Survey English questionnaire.

Multimedia Appendix 2 Sociodemographic traits of San Francisco Health Information National Trends Survey participants.

Multimedia Appendix 3 Unadjusted odds of technology for health purposes.

Multimedia Appendix 4 Adjusted odds of technology for health purposes.

## Abbreviations

aOR: adjusted odds ratio
EHR: electronic health record
HINTS: Health Information National Trends Survey
HITECH: Health Information Technology for Economic and Clinical Health
NCI: National Cancer Institute
NIH: National Institutes of Health
REDCap: Research Electronic Data Capture
SFHINTS: San Francisco Health Information National Trends Survey
TAM: technology acceptance model
