# Supplementary material for: The Use of Technology for Communicating With Clinicians or Seeking Health Information in a Multilingual Urban Cohort: Cross-Sectional Survey
Source: J Med Internet Res. 2020 Apr 6;22(4):e16951. doi: 10.2196/16951 (PMC7171563; doi:10.2196/16951)
Supplement: Multimedia Appendix 1 [file jmir_v22i4e16951_app1.pdf]

SF HINTS ENGLISH Questionnaire

Record ID __________________________________

Thank you again for agreeing to participate in the SF HINTS study. SF HINTS is a study being conducted by researchers at the UCSF Cancer Center and Zuckerberg San Francisco General Hospital to better understand cancer communication needs for the diverse communities of San Francisco. This study is funded by the National Cancer Institute. The goal of this study is to ask questions about how you get information about cancer and your general health. These questions will be used by researchers in the future to determine how to make information and treatment for cancer more accessible to vulnerable populations in San Francisco. The survey will take about 35 min. Do you have a few minutes to talk with me now?

If yes, read:

Thank for taking the time to speak with me. Before I get started, as part of the research process, I want to make sure to acquire formal verbal consent. If you agree to participate in this interview, I will ask you some questions about access to healthcare, cancer screening and demographic information. It is your decision whether you want to participate and you may skip questions at any time.

While there will be no direct benefit to you by participating in this study, your responses will help us learn more about how we can better structure access to their healthcare. A small token of appreciation in the form of a $25 upon completion of the survey is given to participants who complete the survey.

Do you understand and agree to take part in this interview?

If you have any questions about the study, you can call Dr. Urmimala Sarkar at (415) 340-3736 to talk about the study. You can reach the UCSF IRB office at (415) 476-1814, 8 am to 5 pm, Monday through Friday.

We appreciate your time and effort in helping us with this research study.
Do you have any questions before we begin?

Willing to participate in survey?

○ Accept
○ Refuse

A. Health Information Seeking

A1. Have you ever looked for information about health or medical topics from any source?

- ◯ Yes
- ◯ No
- ◯ Don't know
- ◯ Refused

A2. The most recent time you looked for information about health or medical topics, where did you go first? [IF PARTICIPANT HAS MADE MORE THAN ONE SEARCH, ASK FOR THE MOST RECENT SEARCH.]

- ☐ Books
- ☐ Brochures, pamphlets, etc
- ☐ Cancer organization
- ☐ Family
- ☐ Friend/Coworker
- ☐ Health care provider
- ☐ Internet
- ☐ Library
- ☐ Magazines
- ☐ Newspapers
- ☐ Telephone information number (1-800 Number)
- ☐ Complementary or alternative, practitioner
- ☐ Social media site, such as Facebook, PatientsLikeMe, Caring Bridge
- ☐ Other
- ☐ Don't know
- ☐ Refused

A2. If Other, specify

____

A3. If a provider wants to give you more information about your health such as how to prepare for a procedure or education on how to improve your health would you prefer to receive it via:

- ☐ Email
- ☐ Brochures, pamphlets, etc
- ☐ Text message
- ☐ DVD mailed to your home
- ☐ Patient Portal
- ☐ No preference
- ☐ Don't know
- ☐ Refused

B. Health Information Access

B1. Overall, how confident are you that you could get health-related advice or information if you needed it? Would you say. . .

- ◯ Completely confident
- ◯ Very confident
- ◯ Somewhat confident
- ◯ A little confident
- ◯ Not confident at all
- ◯ Don't know
- ◯ Refused

B2. Based on the results of your most recent search for information about health or medical topics, how much do you agree or disagree with the following statements?

	Strongly agree	Somewhat agree	Somewhat disagree	Strongly disagree	DON'T KNOW	REFUSED
a. It took a lot of effort to get the information you needed.	◯	◯	◯	◯	◯	◯
b. You felt frustrated during your search for the information.	◯	◯	◯	◯	◯	◯
c. You were concerned about the quality of the information.	◯	◯	◯	◯	◯	◯
d. The information you found was hard to understand.	◯	◯	◯	◯	◯	◯

B3. Do you currently have a Smart phone such as an iPhone, Android, Blackberry or Windows phone?

- ◯ Yes
- ◯ No

B4. In the past 12 months, have you used any of the following to exchange medical information with a health care professional? Mark all that apply.

- ☐ Email
- ☐ Text message
- ☐ App on a smart phone or mobile device
- ☐ Video conference (e.g., Skype, Facetime, etc.)
- ☐ Social media (e.g., Facebook, Google+, CaringBridge, WeChat, etc.)
- ☐ Instant messaging applications (E.g. WhatsApp, WeChat, etc.)
- ☐ Fax
- ☐ None

B5. Sometimes people use the Internet to connect with other people online through social networks like Facebook or Twitter. This is often called "social media."
In the last 12 months, have you used the Internet for any of the following reasons?

- ☐ Visited a social networking site, such as Facebook or LinkedIn
- ☐ Shared health information on social networking sites, such as Facebook or Twitter
- ☐ Wrote in an online diary or blog (i.e., Web log)
- ☐ Participated in an online forum or support group for people with a similar health or medical issue
- ☐ Watched a health-related video on YouTube
- ☐ None

C. Health Literacy

C1. How often do you need to have someone help you when you read instructions, pamphlets, or other written material from your doctor or pharmacy?

- ◯ Never
- ◯ Rarely
- ◯ Sometimes
- ◯ Often
- ◯ Always
- ◯ Do not know
- ◯ Refused

C2. How confident are you filling out medical forms by yourself?

- ◯ Extremely
- ◯ Quite a bit
- ◯ Somewhat
- ◯ A little
- ◯ Not at all
- ◯ Do not know
- ◯ Refused
- ◯ Does not apply, I don't fill out forms

D. General Health Status (Mental Health)

D1. In general, would you say your health is:

- ◯ Excellent
- ◯ Very good
- ◯ Good
- ◯ Fair
- ◯ Poor

D2. Has a doctor or other health professional ever told you that you had any of the following medical conditions:

	Yes	No
a. Diabetes or high blood sugar?	◯	◯
b. High blood pressure or hypertension?	◯	◯
c. A heart condition such as heart attack, angina, or congestive heart failure?	◯	◯
d. Chronic lung disease, asthma, emphysema, or chronic bronchitis?	◯	◯
e. Arthritis or rheumatism?	◯	◯
f. Depression or anxiety disorder?	◯	◯

D3. Over the past 2 weeks, how often have you been bothered by any of the following problems?

	Not at all	Several days	More than half the days	Nearly every day
a. Little interest or pleasure in doing things	◯	◯	◯	◯
b. Feeling down, depressed or hopeless	◯	◯	◯	◯

D4. About how tall are you without shoes?

____
(a. feet)

____
(b. inches)

D5. About how much do you weigh, in pounds, without shoes?

____
(pounds)

E. Health Behaviors (Tobacco Products, E-cigarettes, Second Hand Smoke, Alcohol Consumption, Physical Activity, Sun Exposure, Hepatitis)

E1. Have you ever smoked at least 100 cigarettes in your entire life?

- ◯ Yes
- ◯ No

E2. Do you now smoke cigarettes:

- ◯ Every day
- ◯ Somedays
- ◯ Not at all
- ◯ Don't know
- ◯ Refused

E3. Do you now use e-cigarettes or other electronics, such as vaping, products every day, some days, or not at all?

- ◯ Every day
- ◯ Somedays
- ◯ Not at all
- ◯ Don't know
- ◯ Refused

E4. During the past 7 days, that is, since last [TODAY DATE OF WEEK], on how many days did you breathe the smoke from someone else who was smoking in an indoor or outdoor public place?

____

- ◯ None
- ◯ Don't know
- ◯ Refused

E5. Now, thinking of your overall drinking in the last 12 months, how often do you usually have any kind of beverage containing alcohol -- whether it is wine, beer, whiskey, coolers, or any other drink? Is it: (IF NECESSARY, ASK: If you had to average it over the last twelve months, how often would it be?)

- ◯ More than once a day
- ◯ Once a day
- ◯ Nearly every day
- ◯ Three or four times a week
- ◯ Once or twice a week
- ◯ Two or three times a month
- ◯ About once a month
- ◯ Less than once a month but at least once a year
- ◯ Less than once a year, or
- ◯ Never had any kind of beverage containing alcohol, or
- ◯ Have you ever had wine?
- ◯ Do not know
- ◯ Refused

E6. During the past month, other than your regular job, did you participate in any physical activities or exercises such as running, golf, gardening, or walking for exercise?

- ◯ Yes
- ◯ No
- ◯ Don't Know
- ◯ Refused

E7. When you go outside on a very sunny day, for more than one hour, how often do you:

a. stay in the shade?

- ◯ Never
- ◯ Rarely
- ◯ Sometimes
- ◯ Most of the time
- ◯ Always
- ◯ Do not know
- ◯ Refused

b. wear a hat that shades your face ears and neck?

- ◯ Never
- ◯ Rarely
- ◯ Sometimes
- ◯ Most of the time
- ◯ Always
- ◯ Do not know
- ◯ Refused

c. wear a long sleeved shirt?

- ◯ Never
- ◯ Rarely
- ◯ Sometimes
- ◯ Most of the time
- ◯ Always
- ◯ Do not know
- ◯ Refused

d. use sunscreen?

- ◯ Never
- ◯ Rarely
- ◯ Sometimes
- ◯ Most of the time
- ◯ Always
- ◯ Do not know
- ◯ Refused

Now I am going to ask you about tests for bloodborne viruses.
Hepatitis is an inflammation of the liver caused by viruses such as hepatitis A, B, or C. The infection can make the skin and eyes turn yellow.

E8. Have you ever had a blood test to check for hepatitis B?

- ◯ Yes
- ◯ No
- ◯ Don't Know
- ◯ Refused

E9. Have you ever had a blood test to check for hepatitis C?

- ◯ Yes
- ◯ No
- ◯ Don't Know
- ◯ Refused

F. Medical Research

F1. Have you ever been asked to participate in a clinical trial or medical research?

- ◯ Yes
- ◯ No
- ◯ Don't Know
- ◯ Refused

F2. Do you think that patients should be asked to take part in medical research?

- ◯ Yes
- ◯ No
- ◯ Don't Know
- ◯ Refused

F3. Have you ever been asked to donate bio specimens (blood, saliva, or other tissue) for the purpose of medical research?

- ◯ Yes
- ◯ No
- ◯ Don't Know
- ◯ Refused

F4. Suppose that you were asked to take part in research study comparing two treatments, both of which were suitable for your illness. Would you be prepared to take part in a study comparing different treatments?

- ◯ Yes
- ◯ No
- ◯ Don't Know
- ◯ Refused

G. Healthcare Access

G1. Is there a place that you USUALLY go to when you are sick or need advice about your health?

- ◯ Yes
- ◯ There is NO place
- ◯ There is MORE THAN ONE place
- ◯ Don't know
- ◯ Refused

G2. What kind of place do you go most often?

- ◯ Clinic or health center
- ◯ Doctor's office or HMO
- ◯ Hospital emergency room
- ◯ Hospital outpatient department
- ◯ Some other place
- ◯ DOESN'T GO TO ONE PLACE MOST OFTEN
- ◯ Don't know
- ◯ Refused

G3. What is the name of the place?

- ◯ Castro-Mission Health Clinic
- ◯ Chinatown Public Health Center
- ◯ Curry Senior Center
- ◯ Family Health Center at ZSFG
- ◯ Housing and Urban Health Clinic
- ◯ Laguna Honda Hospital
- ◯ Larkin Street Medical Center
- ◯ Maxine Hall Health Center
- ◯ Ocean Park Health Center
- ◯ Potrero Hill Health Center
- ◯ San Francisco City Clinic
- ◯ Silver Avenue Family Health Center
- ◯ Southeast Health Center
- ◯ Tom Waddell Health Center
- ◯ Zuckerberg San Francisco General Hospital
- ◯ Glide Health Services
- ◯ Haight-Ashbury Free Medical Clinic
- ◯ Lyon-Martin Women's Health Services
- ◯ Mission Neighborhood Health Center
- ◯ Native American Health Center
- ◯ North East Medical Services
- ◯ San Francisco Free Clinic
- ◯ South of Market Health Center
- ◯ St. Anthony Free Medical Clinic
- ◯ Women's Community Health Center
- ◯ Some other place (please specify)
- ◯ Don't know
- ◯ Refused

G3a. If other, please describe

____

G4. An HIV test checks whether someone has the virus that causes AIDS.

At any time you were seen [by doctor, nurse, or other health professional] were you offered an HIV test?

- ◯ Yes
- ◯ No
- ◯ Don't Know
- ◯ Refused

G5. Do you have any kind of health care coverage, including health insurance, prepaid plans such as HMOs, government plans such as Medicare, or Indian Health Service?

- ◯ Yes
- ◯ No
- ◯ Don't Know
- ◯ Refused

G6. What is the primary source of your health care coverage? Is it...PLEASE READ

- ◯ A plan purchased through an employer or union (includes plans purchased through another person's employer)
- ◯ A plan that you or another family member buys on your own
- ◯ Medicare
- ◯ Medicaid or other state program
- ◯ TRICARE (formerly CHAMPUS), VA, or Military
- ◯ Alaska Native, Indian Health Service, Tribal Health Services
- ◯ Some other source
- ◯ None (no coverage)
- ◯ Don't know
- ◯ Refused

G7. In the past 12 months was there a time when you needed to see a doctor, but could not because of cost?

- ◯ Yes
- ◯ No
- ◯ Don't Know
- ◯ Refused

G8. What are the main reasons you did not get medical care? Check all that apply.

- ☐ Cost [Include no insurance]
- ☐ Distance
- ☐ Office wasn't open when I could get there
- ☐ Too long a wait for an appointment
- ☐ No care available for child, disabled, or elderly person I care for
- ☐ No transportation
- ☐ No access for people with disabilities
- ☐ The medical provider didn't speak my language
- ☐ I could not get time off of work to go
- ☐ Other (specify)
- ☐ Don't know
- ☐ Refused

G8a. If other, please describe

____

G9. In the past 12 months, not counting times you went to an emergency room, how many times did you go to a doctor, nurse, or other health professional to get care for yourself?

- ◯ None
- ◯ 1 time
- ◯ 2 times
- ◯ 3 times
- ◯ 4 times
- ◯ 5-9 times
- ◯ 10 or more times

G10. Overall, how would you rate the quality of health care you received in the past 12 months?

- ◯ Excellent
- ◯ Very good
- ◯ Good
- ◯ Fair
- ◯ Poor
- ◯ I did not receive healthcare in the last 12 months.

H. Sociodemographics

We are about half way through the survey.

H1. What is your age?

◯ 18
◯ 19
◯ 20
◯ 21
◯ 22
◯ 23
◯ 24
◯ 25
◯ 26
◯ 27
◯ 28
◯ 29
◯ 30
◯ 31
◯ 32
◯ 33
◯ 34
◯ 35
◯ 36
◯ 37
◯ 38
◯ 39
◯ 40
◯ 41
◯ 42
◯ 43
◯ 44
◯ 45
◯ 46
◯ 47
◯ 48
◯ 49
◯ 50
◯ 51
◯ 52
◯ 53
◯ 54
◯ 55
◯ 56
◯ 57
◯ 58
◯ 59
◯ 60
◯ 61
◯ 62
◯ 63
◯ 64
◯ 65
◯ 66
◯ 67
◯ 68
◯ 69
◯ 70
◯ 71
◯ 72
◯ 73
◯ 74
◯ 75
◯ 76
◯ 77
◯ 78
◯ 79
◯ 80
◯ 81
◯ 82
◯ 83
◯ 84
◯ 85
◯ 86
◯ 87

- ◯ 88
- ◯ 89
- ◯ 90
- ◯ Don't Know
- ◯ Refused

(Years old)

H2. Are you of Hispanic, Latino/a, or Spanish Origin? If Yes, are you-

- ☐ Mexican, Mexican American, Chicano/a
- ☐ Puerto Rican
- ☐ Cuban
- ☐ Other
- ☐ No
- ☐ Don't know
- ☐ Refused

H3. Which of the following describes your race? You can select as many as apply. Are you
Mark all that apply

- ☐ White or Caucasian
- ☐ Black or African American
- ☐ American Indian or Alaska Native
- ☐ Asian Indian
- ☐ Chinese
- ☐ Filipino
- ☐ Japanese
- ☐ Korean
- ☐ Vietnamese
- ☐ Other Asian
- ☐ Native Hawaiian
- ☐ Guamanian or Chamorro
- ☐ Samoan
- ☐ Other Pacific Islander
- ☐ Other
- ☐ Don't know/Not sure
- ☐ Refused

H3a. If Other, please describe

____

H4. How well do you speak English? Would you say:

- ◯ Not at all
- ◯ Not well
- ◯ Well
- ◯ Very Well
- ◯ Poor

H5. What is your marital status? Would you say:
NOTE: Living as married is the legal basis for common law marriage: two people cohabitating together, regardless of romantic relationship.

- ◯ Married
- ◯ Domestic partnership
- ◯ Divorced
- ◯ Widowed
- ◯ Seperated, or
- ◯ Single, never been married?
- ◯ Living as married
- ◯ Don't Know
- ◯ Refused

H6. What is the highest grade or level of schooling you completed?

- ◯ Less than 8 years
- ◯ 8 through 11 years
- ◯ 12 years or completed high school
- ◯ Post high school training other than college (vocational or technical)
- ◯ Some college
- ◯ College graduate
- ◯ Postgraduate
- ◯ Don't know
- ◯ Refused

H7. Do you currently rent or own your home?
NOTE: Home is defined as the place where you live most of the time/the majority of the year.

◯ Own
◯ Rent
◯ Occupied without paying monetary rent
◯ Don't Know/Not sure
◯ Refused

H8. What is the ZIP Code where you live?

____

H9. Were you born in the United States?

◯ Yes
◯ No
◯ Don't Know
◯ Refused

H10. In what year did you come to live in the United States?

____

H11. Are you currently...?

◯ Employed
◯ Unemployed
◯ Homemaker
◯ Student
◯ Retired
◯ Disabled
◯ Employed - part time
◯ Other
◯ Don't know
◯ Refused

H11a. If other, specify

____

H12. Including yourself, how many people live in your household?

____

H13. Thinking about members of your family living in this household, what is your combined annual income, meaning the total pre-tax income from all sources earned in the past year? Is it ....

◯ Less than $10,000
◯ $10,000 to under $15,000
◯ $15,000 to under $20,000
◯ $20,000 to under $35,000
◯ $35,000 to under $50,000
◯ $50,000 to under $75,000
◯ $75,000 to under $100,000
◯ $100,000 to under $200,000
◯ $200,000 or more
◯ Don't know
◯ Refused

H14. Which one of these phrases comes closest to your own feelings about your household?s income these days?

◯ Living comfortably on present income
◯ Getting by on present income
◯ Finding it difficult on present income
◯ Finding it very difficult on present income

H15. What is your current gender identity?

◯ Male
◯ Female
◯ Female-to-Male (FTM)/Transgender Male/Trans Man
◯ Male-to-Female (MTF)/Transgender Female/ Trans Woman
◯ Genderqueer, neither exclusively male nor female
◯ Additional Gender Category/(or Other) (Please Specify)
◯ Decline to answer, please explain why

H15a. Additional Gender Category/(or Other) (Please Specify); Decline to answer, please explain why

____

H16. What sex were you assigned at birth on your original birth certificate (check only one)?

- ◯ Male
- ◯ Female
- ◯ Decline to answer, please explain why

H16a. Decline to answer, please explain why ______________________

H17. How do you describe your sexual orientation or sexual identity

- ◯ Straight / Heterosexual
- ◯ Bisexual
- ◯ Gay / Lesbian / Same-Gender Loving
- ◯ Questioning / Unsure
- ◯ Not listed (Please Specify): __________
- ◯ Decline to answer

If other, please describe: ______________________

I. Housing Stability

I1. In the past year, have you slept in the street, homeless shelter, or place not ordinarily used as a sleeping accommodation (bus shelter, storefront) because you had no other place to stay?

- ◯ Yes
- ◯ No
- ◯ Don't Know
- ◯ Refused

I2. In the past YEAR, have you stayed/doubled up with friends or family, because you haven?t had another place to stay?

- ◯ Yes
- ◯ No
- ◯ Don't Know
- ◯ Refused

I3. In the past YEAR, have you lived in a single-room occupancy (SRO) hotel?

- ◯ Yes
- ◯ No
- ◯ Don't Know
- ◯ Refused

J. Beliefs about Cancer

Think about cancer in general when answering the questions in this section

J1. How much do you agree or disagree with each of the following statements?

	Strongly agree	Somewhat agree	Somewhat disagree	Strongly disagree
a. It seems like everything causes cancer. Would you say you?	◯	◯	◯	◯
b. There's not much you can do to lower your chances of getting cancer. Would you say you?	◯	◯	◯	◯
c. There are so many different recommendations about preventing cancer, it's hard to know which ones to follow. Would you say you?	◯	◯	◯	◯

d. Cancer is most often caused by a person's behavior or lifestyle.	◯	◯	◯	◯
e. When I think about cancer, I automatically think about death.	◯	◯	◯	◯

J2. Have you ever been diagnosed as having cancer?

- ◯ Yes
- ◯ No

J3. What type of cancer did you have? Mark all that apply.

- ☐ Bladder cancer
- ☐ Bone cancer
- ☐ Breast cancer
- ☐ Cervical cancer (cancer of the cervix)
- ☐ Colon cancer
- ☐ Endometrial cancer (cancer of the uterus)
- ☐ Head and neck cancer
- ☐ Hodgkin's lymphoma
- ☐ Leukemia/Blood cancer
- ☐ Liver cancer
- ☐ Lung cancer
- ☐ Melanoma
- ☐ Non-Hodgkin lymphoma
- ☐ Oral cancer
- ☐ Ovarian cancer
- ☐ Pancreatic cancer
- ☐ Pharyngeal (throat) cancer
- ☐ Prostate cancer
- ☐ Rectal cancer
- ☐ Renal (kidney) cancer
- ☐ Skin cancer, non-melanoma
- ☐ Stomach cancer
- ☐ Other

J4. Have any of your family members ever had cancer?

- ◯ Yes
- ◯ No
- ◯ Not sure

K. Awareness of Cancer Risk

K1. Compared to other people your age, how likely are you to get cancer in your lifetime? Would you say you are

- ◯ Very unlikely
- ◯ Unlikely
- ◯ Neither unlikely nor likely
- ◯ Likely
- ◯ Very likely
- ◯ Don't know
- ◯ Refused

K2. I'd rather not know my chance of getting cancer.

- ◯ Strongly agree
- ◯ Somewhat agree
- ◯ Somewhat disagree
- ◯ Strongly disagree
- ◯ Don't know
- ◯ Refused

L. Breast and Cervical Cancer Screening

If male go to the next section.

L1. A mammogram is an x-ray of each breast to look for breast cancer. Have you ever had a mammogram?

- ◯ Yes
- ◯ No
- ◯ Don't Know
- ◯ Refused

L2. How long has it been since you had your last mammogram?

- ◯ Within the past year (anytime less than 12 months ago)
- ◯ Within the past 2 years (1 year but less than 2 years ago)
- ◯ Within the past 3 years (2 years but less than 3 years ago)
- ◯ Within the past 5 years (3 years but less than 5 years ago)
- ◯ 5 or more years ago
- ◯ Don't Know
- ◯ Refused

L3. A Pap test is a test for cancer of the cervix. Have you ever had a Pap test?

- ◯ Yes
- ◯ No
- ◯ Don't Know
- ◯ Refused

L4. How long has it been since you had your last Pap test?

- ◯ Within the past year (anytime less than 12 months ago)
- ◯ Within the past 2 years (1 year but less than 2 years ago)
- ◯ Within the past 3 years (2 years but less than 3 years ago)
- ◯ Within the past 5 years (3 years but less than 5 years ago)
- ◯ 5 or more years ago
- ◯ Don't Know
- ◯ Refused

Now, I would like to ask you about the Human Papillomavirus (Pap.uh.loh.muh virus) or HPV test.

L5. How many HPV shots did you receive?

- ◯ 0
- ◯ 1
- ◯ 2
- ◯ All shots (3)
- ◯ Don't know/not sure
- ◯ Refused

L6. Have you had a hysterectomy? Read if necessary: A hysterectomy is an operation to remove the uterus (womb).

- ◯ Yes
- ◯ No
- ◯ Don't Know
- ◯ Refused

M. Prostate Cancer Screening

If respondent is < 39 years of age, or is female, go to next section.

Now, I will ask you some questions about prostate cancer screening. A Prostate-Specific Antigen test, also called a PSA test is a blood test used to check men for prostate cancer.

M1. Have you ever had a PSA test?

- ◯ Yes
- ◯ No

M2. How long has it been since you had your last PSA test?

- ◯ Within the past year (anytime less than 12 months ago)
- ◯ Within the past 2 years (1 year but less than 2 years ago)
- ◯ Within the past 3 years (2 years but less than 3 years ago)
- ◯ Within the past 5 years (3 years but less than 5 years ago)
- ◯ 5 or more years ago
- ◯ Don't Know
- ◯ Refused

M3. Has a MD or Nurse ever talked to you about whether you should or should not have a PSA test?

- ◯ Yes
- ◯ No

M4. What was the MAIN reason you had this PSA test, was it ?

- ◯ Part of a routine exam
- ◯ Because of a prostate problem
- ◯ Because of a family history of prostate cancer
- ◯ Because you were told you had prostate cancer
- ◯ Some other reason
- ◯ Don't Know
- ◯ Refused

N. Colorectal Cancer Screening

If respondent is < 49 years of age, go to next section.

If respondent is < 49 years of age, go to next section.
The next questions are about colorectal cancer screening.

N1. A blood stool test, (sometimes called FOBT or FIT) is a special test done at home to determine whether the stool contains blood. It is used to look for small amounts of blood in your stool that could come from colon cancers or other health problems. Have you ever had a blood stool test to screen for cancer?

- ◯ Yes
- ◯ No
- ◯ Don't Know
- ◯ Refused

N2. How long has it been since you had your last blood stool test using a home kit?

- ◯ Within the past year (anytime less than 12 months ago)
- ◯ Within the past 2 years (1 year but less than 2 years ago)
- ◯ 2 or more years
- ◯ Don't Know
- ◯ Refused

N3. Sigmoidoscopy and colonoscopy are exams in which a tube is inserted in the rectum to view the colon for signs of cancer or other health problems. Have you ever had either of these exams?

- ◯ Yes
- ◯ No
- ◯ Don't Know
- ◯ Refused

N4. For a SIGMOIDOSCOPY, a flexible tube is inserted into the rectum to look for problems. A COLONOSCOPY is similar, but uses a longer tube, and you are usually given medication through a needle in your arm to make you sleepy and told to have someone else drive you home after the test. Was your MOST RECENT exam a sigmoidoscopy or a colonoscopy?

- ◯ Sigmoidoscopy
- ◯ Colonoscopy
- ◯ Don't Know
- ◯ Refused

N5. How long has it been since you had your last sigmoidoscopy or colonoscopy?

- ◯ Within the past 10 years
- ◯ 10 or more years ago
- ◯ Don't Know
- ◯ Refused

O. Knowledege of Cancer Screening

Just two more questions.

O1. At what age are most women supposed to start having mammograms?
NOTE: IF RESPONDENT SAYS, "WHEN A DOCTOR SAYS TO," ASK FOR AN ESTIMATE OF THE AGE.

____

O2. At what age are most people supposed to start doing home blood stool tests, having a sigmoidoscopy or having a colonoscopy? NOTE: IF RESPONDENT SAYS, "WHEN A DOCTOR SAYS TO," ASK FOR AN ESTIMATE OF THE AGE.

- ◯ 1
- ◯ 2
- ◯ 3
- ◯ 4
- ◯ 5
- ◯ 6
- ◯ 7
- ◯ 8
- ◯ 9
- ◯ 10
- ◯ 11
- ◯ 12
- ◯ 13
- ◯ 14
- ◯ 15
- ◯ 16
- ◯ 17
- ◯ 18
- ◯ 19
- ◯ 20
- ◯ 21
- ◯ 22
- ◯ 23
- ◯ 24
- ◯ 25
- ◯ 26
- ◯ 27
- ◯ 28
- ◯ 29
- ◯ 30
- ◯ 31
- ◯ 32
- ◯ 33
- ◯ 34
- ◯ 35
- ◯ 36
- ◯ 37
- ◯ 38
- ◯ 39
- ◯ 40
- ◯ 41
- ◯ 42
- ◯ 43
- ◯ 44
- ◯ 45
- ◯ 46
- ◯ 47
- ◯ 48
- ◯ 49
- ◯ 50
- ◯ 51
- ◯ 52
- ◯ 53
- ◯ 54
- ◯ 55
- ◯ 56
- ◯ 57
- ◯ 58
- ◯ 59
- ◯ 60
- ◯ 61
- ◯ 62
- ◯ 63
- ◯ 64
- ◯ 65
- ◯ 66
- ◯ 67
- ◯ 68
- ◯ 69
- ◯ 70

◯ 71
◯ 72
◯ 73
◯ 74
◯ 75
◯ 76
◯ 77
◯ 78
◯ 79
◯ 80
◯ 81
◯ 82
◯ 83
◯ 84
◯ 85
◯ 86
◯ 87
◯ 88
◯ 89
◯ 90
◯ 91
◯ 92
◯ 93
◯ 94
◯ 95
◯ 96
◯ 97
◯ 98
◯ 99
◯ 100
◯ 101
◯ 102
◯ 103
◯ 104
◯ 105
◯ 106
◯ 107
◯ 108
◯ 109
◯ 110
◯ 111
◯ 112
◯ 113
◯ 114
◯ 115
◯ 116
◯ 117
◯ 118
◯ 119
◯ 120
◯ 121
◯ 122
◯ 123
◯ 124
◯ 125
◯ 126
◯ 127
◯ 128
◯ 129
◯ 130
◯ Don't Know
◯ Refused
(Years old)

SURVEY COMPLETE. Return device to survey administrator. __________________________________

Surveyor Initials
