# Supplementary material for: The Use of Technology for Communicating With Clinicians or Seeking Health Information in a Multilingual Urban Cohort: Cross-Sectional Survey
Source: J Med Internet Res. 2020 Apr 6;22(4):e16951. doi: 10.2196/16951 (PMC7171563; doi:10.2196/16951)
Supplement: Multimedia Appendix 2 [file jmir_v22i4e16951_app2.docx]

**Appendix 2**. Sociodemographic Characteristic Traits of SFHINTS Participants^a^ and San Francisco County^b^

| **Characteristic Trait** | **San Francisco (n=864,263)** | **All**  **(n = 1027)** | **English**  **(n = 514)** | **Spanish**  **(n = 256)** | **Chinese**  **(n = 257)** |
| --- | --- | --- | --- | --- | --- |
| Age | 38.3 ^b^ |  |  |  |  |
| 18-34 years old |  | 281 (28%) | 166 (32%) | 52 (20%) | 63 (25%) |
| 35-49 years old |  | 261 (25%) | 122 (24%) | 96 (38%) | 43 (17%) |
| 50-64 years old |  | 305 (30%) | 177 (34%) | 75 (29%) | 53 (21%) |
| 65+ years old | 128,384 (15%) | 180 (18%) | 49 (10%) | 33 (13%) | 98 (38%) |
|  |  |  |  |  |  |
| Female (n, %) | 423,630 (49%) | 536 (52%) | 286 (56%) | 122 (48%) | 128 (50%) |
|  |  |  |  |  |  |
| Race / Ethnicity (n, %) ^*^ |  |  |  |  |  |
| Non-Hispanic White | 353,000 (41%) | 44 (4%) | 43 (8%) | 1 (0.4%) | 0 (0%) |
| Non-Hispanic Black | 43,961 (5%) | 243 (24%) | 242 (47%) | 1 (0.4%) | 0 (0%) |
| Latinx | 131,949 (15%) | 365 (36%) | 115 (22%) | 250 (98%) | 0 (0%) |
| Non-Hispanic Asian / Pacific-Islander | 295,619 (34%) | 317 (31%) | 58 (11%) | 2 (0.8%) | 257 (100%) |
| Other | 38,299 (4%) | 58 (6%) | 56 (11%) | 2 (0.8%) | 0 (0%) |
|  |  |  |  |  |  |
| Limited English Proficient (n, %) ^*^ | 170,041 (21%) | 344 (34%) | 27 (5%) | 147 (57%) | 170 (66%) |
|  |  |  |  |  |  |
| Education (n, %) |  |  |  |  |  |
| Less than high school education | 82,618 (12%) | 236 (23%) | 61 (12%) | 88 (35%) | 87 (34%) |
| High school graduate / equivalent | 84,072 (12%) | 305 (30%) | 160 (31%) | 75 (29%) | 70 (27%) |
| Some college / vocational training | 135,318 (20%) | 284 (28%) | 167 (32%) | 59 (23%) | 58 (23%) |
| College graduate | 381,066 (56%) | 186 (18%) | 115 (22%) | 31 (12%) | 40 (16%) |
|  |  |  |  |  |  |
| Limited Health Literacy (n, %) ^*^ | -- ^b^ | 440 (43%) | 187 (36%) | 107 (42%) | 146 (57%) |
|  |  |  |  |  |  |
| Fair/poor health status (n, %) ^*^ | -- ^b^ | 681 (66%) | 375 (73%) | 167 (65%) | 139 (54%) |
|  |  |  |  |  |  |
| Smartphone ownership (n, %) ^*^ | -- ^b^ | 791 (77%) | 398 (77%) | 211 (82%) | 182 (71%) |
|  |  |  |  |  |  |
| Type of Clinic for Usual Source of Care ^*^ | -- ^b^ |  |  |  |  |
| No usual source of care |  | 178 (17%) | 82 (16%) | 34 (13%) | 62 (24%) |
| Independent safety net clinic |  | 148 (17%) | 49 (10%) | 35 (16%) | 58 (23%) |
| Integrated safety net health system |  | 378 (37%) | 196 (38%) | 91 (43%) | 75 (28%) |
| Private clinic / community hospital |  | 120 (12%) | 56 (11%) | 26 (11%) | 36 (14%) |
| Academic tertiary medical center |  | 31 (3%) | 22 (4%) | 6 (2%) | 2 (1%) |
| Integrated payer and provider |  | 125 (12%) | 75 (15%) | 31 (13%) | 16 (6%) |

^a^ In the SFHINTS cohort, data was missing for 3 participants for gender; 17 participants for education, 22 participants for health literacy; 47 for type of clinic; Data for San Francisco County reported based on data available from US Census Bureau 2013-2017 American Community Survey 5-Year Estimates.

^b^ Citywide data from the 2017 American Community Survey. These data do not include health literacy, health status, smartphone ownership, or type of clinic. The American Community Survey does not report the same age breakdown as used in this study so median age is reported instead and population 65+. Education percentages are reported out of population 25 years +.

^*^ significant differences among all groups (p < 0.05)
