# Supplementary material for: The Use of Technology for Communicating With Clinicians or Seeking Health Information in a Multilingual Urban Cohort: Cross-Sectional Survey
Source: J Med Internet Res. 2020 Apr 6;22(4):e16951. doi: 10.2196/16951 (PMC7171563; doi:10.2196/16951)
Supplement: Multimedia Appendix 3 [file jmir_v22i4e16951_app3.docx]

Appendix 3. Unadjusted odds of using technology for health purposes

| Predictor | Email with Clinician uOR [95% CI] | SMS with Clinician  uOR [95% CI] | App with  Clinician  uOR [95% CI] | Support Group  uOR [95% CI] | Online Videos  uOR [95% CI] |
| --- | --- | --- | --- | --- | --- |
| Age ^a^ |  |  |  |  |  |
| 35-49 years | 0.89 [0.45, 0.76] * | 1.38 [0.60, 3.16] | 0.26 [0.11, 0.65] * | 0.91 [0.31, 2.68] | 0.65 [0.33, 1.28] |
| 50-64 years | 0.77 [0.38, 1.58] | 1.35 [0.56, 3.25] | 0.22 [0.10, 0.51] * | 0.48 [0.13, 1.78] | 0.43 [0.22, 0.86] * |
| 65+ years | 0.31 [0.12, 0.80] * | 0.81 [0.27, 2.42] * | 0.07 [0.03, 0.20] * | 0.48 [0.10, 2.20] | 0.16 [0.07, 0.39] * |
| Gender |  |  |  |  |  |
| Female | 1.78 [1.07, 2.98] * | 1.16 [0.59, 2.26] | 1.13 [0.49, 2.62] | 0.71 [0.29, 1.77] | 1.02 [0.60, 1.75] |
| Race/ethnicity **^a^** |  |  |  |  |  |
| Black | 0.41 [0.19, 0.90] * | 2.00 [0.71, 5.61] | 1.43 [0.43, 4.87] | 1.38 [0.44, 4.35] | 0.94 [0.42, 2.08] |
| Hispanic | 0.53 [0.24, 1.13] | 1.50 [0.54, 4.19] | 1.48 [0.45, 4.90] | 1.06 [0.34, 3.27] | 1.37 [0.63, 2.96] |
| Asian | 0.31 [0.14, 0.67] * | 0.43 [0.15, 1.26] | 0.64 [0.18, 2.23] | 0.94 [0.30, 2.98] | 0.99 [0.45, 2.17] |
| Other | 0.73 [0.27, 2.01] | 1.65 [0.48, 5.70] | 2.24 [0.53, 9.52] | 1.99 [0.45, 8.77] | 2.39 [0.87, 6.58] |
| Limited English proficient | 0.25 [0.14, 0.44] * | 0.67 [0.35, 1.28] | 0.31 [0.16, 0.60] * | 0.38 [0.18, 0.79] * | 0.46 [0.29, 0.75] * |
| Education **^a^** |  |  |  |  |  |
| Less than high school education | 0.11 [0.05, 0.24] * | 0.56 [0.26, 1.20] | 0.38 [ 0.13, 1.16] | 0.44 [0.17, 1.15] | 0.30 [0.16, 0.58] * |
| High school graduate / equivalent | 0.25 [0.13, 0.48] * | 0.80 [0.38, 1.66] | 0.35 [0.14, 0.84] * | 0.27 [0.11, 0.66] * | 0.52 [0.29, 0.94] * |
| Some college / vocational training | 0.55 [0.30, 1.02] | 0.82 [0.40, 1.72] | 1.25 [0.54, 2.90] | 0.45 [0.18, 1.13] | 0.89 [0.49, 1.62] |
| Limited health literacy | 2.50 [1.46, 4.25] * | 1.20 [0.61, 2.36] | 1.48 [0.65, 3.36] | 1.63 [0.67, 3.96] | 2.08 [1.26, 3.42] |
| Fair/poor health | 0.58 [0.31, 1.07] | 1.47 [0.71, 3.05] | 0.65 [0.25, 1.68] | 0.42 [ 0.15, 1.15] | 0.39 [0.22, 0.68] * |
| **Primary predictor variables of interest** |  |  |  |  |  |
| No smartphone | 0.25 [0.12, 0.52] * | 0.41 [0.17, 0.99] * | 0.325 [0.13, 0.84] * | 0.10 [0.04, 0.26] * | 0.14 [0.07, 0.25] * |
| Language **^a^** |  |  |  |  |  |
| Spanish | 0.48 [0.27, 0.85] * | 0.85 [0.46, 1.57] | 0.51 [0.25, 1.04] | 0.46 [0.20, 1.06] | 0.76 [0.42, 1.36] |
| Chinese | 0.29  [0.17, 0.49] * | 0.23  [0.12, 0.44] * | 0.22  [0.10, 0.47]* | 0.64  [0.29, 1.42] | 0.52  [0.32, 0.84] * |
| Type of clinic for usual source of care **^a^** |  |  |  |  |  |
| Non-integrated safety net | 1.10 [0.43, 2.80] | 0.56 [0.25, 1.27] | 0.74 [0.27, 2.00] | 0.54 [0.19, 1.52] | 0.91 [0.42, 1.96] |
| Integrated safety-net | 2.03  [1.04, 3.98] * | 3.26  [1.44, 7.38] * | 1.11  [0.44, 2.83] | 0.83  [0.27, 2.50] | 1.59  [0.83, 3.05] |
| Private clinic / community hospital | 1.15  [0.45, 2.94] | 0.69  [0.26,1.83] | 1.17  [0.42, 3.29] | 0.37  [0.11, 1.25] | 2.85  [1.13, 7.19] * |
| Academic tertiary medical center | 14.10  [4.58, 43.38] * | 4.54  [1.03, 20.02] * | 9.09  [2.05, 40.28] * | 1.36  [0.21, 8.72] | 3.51  [0.90, 13.72] |
| Integrated payer and provider | 4.46  [1.71, 11.64] * | 0.83 [0.30, 2.28] | 3.92  [1.22, 12.58] * | 2.14 [0.57, 8.08] | 2.66  [1.10, 6.43] * |

**^a^** The reference variable for each variable is: age (18-34), race/ethnicity (White), education (at least college graduate), language (English), type of clinic (no usual source of care).

**^b^** All odds ratios are weighted but unadjusted within this table.

**^*^** p < 0.05
