# Supplementary material for: The Use of Technology for Communicating With Clinicians or Seeking Health Information in a Multilingual Urban Cohort: Cross-Sectional Survey
Source: J Med Internet Res. 2020 Apr 6;22(4):e16951. doi: 10.2196/16951 (PMC7171563; doi:10.2196/16951)
Supplement: Multimedia Appendix 4 [file jmir_v22i4e16951_app4.docx]

**Appendix 4.** Adjusted odds of using technology for health purposes **^a^**

| Predictor | Email with Clinician aOR [95% CI] | SMS with Clinician  aOR [95% CI] | App with  Clinician  aOR [95% CI] | Support Group  aOR [95% CI] | Online Videos  aOR [95% CI] |
| --- | --- | --- | --- | --- | --- |
| Age ^a^ |  |  |  |  |  |
| 35-49 years | 0.97 [0.45, 2.07] | 1.81 [0.76, 4.31] | 0.31 [0.12, 0.82] * | 1.45 [0.49, 4.24] | 1.10 [0.56, 2.18] |
| 50-64 years | 0.88 [0.41, 1.90] | 2.04 [0.81, 5.11] | 0.17 [0.06, 0.50] * | 1.11 [0.31, 4.00] | 0.70 [0.34, 1.45] |
| 65+ years | 0.38 [0.10, 1.43] | 0.96 [0.25, 3.68] | 0.06 [0.01, 0.23] * | 2.03 [0.31, 13.24] | 0.26 [0.09, 0.77] * |
| Gender |  |  |  |  |  |
| Female | 2.22 [1.20, 4.10] * | 1.10 [0.55, 2.15] | 0.93 [0.39, 2.25] | 0.67 [0.26, 1.69] | 1.02 [0.61, 1.71] |
| Race/ethnicity **^a^** |  |  |  |  |  |
| Black | 0.54 [0.23, 1.26] | 2.85 [0.90, 9.07] | 2.23 [0.78, 6.41] | 4.14 [0.85, 20.08] | 1.01 [0.41, 2.55] |
| Hispanic | 0.80 [0.31, 2.07] | 2.49 [0.66, 9.41] | 2.06 [0.66, 6.40] | 4.91 [0.91, 26.53] | 1.36 [0.46, 4.07] |
| Asian | 0.48 [0.17, 1.33] | 1.70 [0.49, 5.93] | 1.37 [0.32, 5.50] | 1.16 [0.17, 7.89] | 1.01 [0.31, 3.26] |
| Other | 0.60 [0.19, 1.90] | 2.98 [0.89, 10.01] | 1.71 [0.47, 6.21] | 4.44 [0.65, 30.49] | 2.52 [0.88, 7.21] |
| Limited English proficient | 0.97 [0.42, 2.26] | 1.73 [0.71, 4.21] | 2.08 [0.85, 5.10] | 0.75 [0.27, 2.11] | 1.88 [0.91, 3.85] |
| Education **^a^** |  |  |  |  |  |
| Less than high school education | 0.22 [0.08, 0.59] * | 0.76 [0.32, 1.84] | 1.07 [0.34, 3.32] | 0.94 [0.26, 3.41] | 0.88 [0.37, 2.09] |
| High school graduate / equivalent | 0.40 [0.19, 0.85] * | 0.99 [0.45, 2.16] | 0.57 [0.22, 1.44] | 0.32 [0.11, 0.91] * | 0.75 [0.39, 1.44] |
| Some college / vocational training | 0.67 [0.36, 1.24] * | 0.90 [0.40, 2.02] | 1.47 [0.71, 3.05] | 0.43 [0.15, 1.20] | 1.03 [0.56, 1.90] |
| Limited health literacy | 1.24 [0.68, 2.25] | 1.11 [0.56, 2.18] | 0.74 [0.32, 1.68] | 0.89 [0.33, 2.38] | 1.56 [0.86, 2.81] |
| Fair/poor health | 0.99 [0.41, 1.94] | 1.68 [0.72, 3.91] | 0.98 [0.48, 1.98] | 0.43 [0.17, 1.11] | 0.54 [0.30, 0.98] * |
| **Primary predictor variables of interest** |  |  |  |  |  |
| No smartphone | 0.61 [0.25, 1.48] | 0.27 [0.13, 0.56] * | 1.12 [0.42, 2.99] | 0.14 [0.04, 0.55] * | 0.31 [0.15, 0.64] * |
| Language **^a^** |  |  |  |  |  |
| Spanish | 0.69 [0.28, 1.69] | 0.51 [0.16, 1.62] | 0.34 [0.16, 0.75] * | 0.30 [0.10, 0.92] * | 0.66 [0.21, 2.02] |
| Chinese | 0.97 [0.41, 2.30] | 0.25 [0.08, 0.79] * | 0.32 [0.10, 1.03] | 2.01 [0.50, 8.06] | 1.00 [0.41, 2.41] |
| Type of clinic for usual source of care **^a^** |  |  |  |  |  |
| Non-integrated safety net | 1.02 [0.39, 2.66] | 0.64 [0.25, 1.63] | 1.12 [0.36, 3.54] | 0.55 [0.17, 1.81] | 0.68 [0.27, 1.69] |
| Integrated safety-net | 2.36 [1.08, 5.12] * | 2.96 [1.25, 7.04] * | 1.60 [0.57, 4.54] | 0.47 [0.14, 1.57] | 1.35 [0.65, 2.83] |
| Private clinic / community hospital | 1.02 [0.33, 3.15] | 0.72 [0.22, 2.33] | 1.41 [0.31, 6.42] | 0.19 [0.03, 1.08] | 2.65 [1.08, 6.51] * |
| Academic tertiary medical center | 9.08  [2.46, 33.5] * | 3.39  [0.57, 20.19] | 12.41  [2.76, 55.89] * | 0.70 [0.11, 4.28] | 2.50 [0.75, 8.29] |
| Integrated payer and provider | 2.68 [0.99, 7.27] | 0.78 [0.27, 2.26] | 4.81 [1.44, 16.05] * | 1.20 [0.30, 4.80] | 1.79 [0.74, 4.32] |

**^a^** The reference variable for each variable is: age (18-34), race/ethnicity (White), education (at least college graduate), language (English), type of clinic (no usual source of care).

**^b^** All odds ratios are adjusted within this table.

**^*^** p < 0.05
